# RGG-motif self-association regulates eIF4G-binding translation repressor protein Scd6

**DOI:** 10.1080/15476286.2019.1621623

**Published:** 2019-06-12

**Authors:** Gopalakrishna Poornima, Ravishankar Mythili, Priyabrata Nag, Sabnam Parbin, Praveen Kumar Verma, Tanweer Hussain, Purusharth I Rajyaguru

**Affiliations:** a Department of Biochemistry, Indian Institute of Science, Bangalore, India; b Department of Biology, University of Western Ontario, London, Canada; c Department of Molecular Reproduction, Development and Genetics, Indian Institute of Science, Bangalore, India

**Keywords:** Translation control, mRNA fate, eIF4G, RGG-motif proteins, self-association, Scd6 and Sbp1

## Abstract

Regulation of mRNA translation plays a key role in the control of gene expression. Scd6, a conserved RGG-motif containing protein represses translation by binding to translation initiation factor eIF4G1. Here we report that Scd6 binds itself in RGG-motif dependent manner and self-association regulates its repression activity. Scd6 self-interaction competes with eIF4G1 binding and methylation of Scd6 RGG-motif by Hmt1 negatively affects self-association. Results pertaining to Sbp1 indicate that self-association could be a general feature of RGG-motif containing translation repressor proteins. Taken together, our study reveals a mechanism of regulation of eIF4G-binding RGG-motif translation repressors.

## Introduction

Control of mRNA translation and decay is important for changes in gene expression in various cellular processes. Translation control is critical during cell division, maturation, early embryonic development, learning, memory and synaptic plasticity [1]. Transcription is largely silent during meiosis and gene expression at the level of translation becomes crucial [2]. A recent study suggests that 91% of the mRNAs that undergo gene-specific regulation during mitosis are translationally repressed, highlighting the importance of translation repression [3]. Misregulated translation underlies a variety of human diseases including cancers, neurodegenerative and metabolic disorders [4–6]. One of the much-studied examples is the implications of eIF4E overexpression and misregulation in several cancers [7].

Translation initiation is considered to be the most regulated step of translation. Regulatory mechanisms have been reported for almost all major steps in translation initiation [8,9]. Formation of the cap-binding complex is a key step during initiation that involves the association of the eIF4E-eIF4G complex with the mRNA cap along with other proteins. eIF4G is a conserved scaffolding protein that apart from binding eIF4E, provides a platform to recruit other initiation factors such as polyA-binding protein (Pab1) and eIF4A (a DEAD-box RNA helicase) [10,11]. Given the importance of cap-binding complex in translation initiation, it is not surprising that many translation control mechanisms target this complex especially eIF4E [12,13]. Many studies have reported upregulation of the cap-binding complex components including eIF4G in cancers, which is consistent with the idea that deregulated translation is a hallmark of cancer [14].

Recent reports have highlighted the theme that scaffolding protein like eIF4G can differentially impact mRNA fate depending on the factors it recruits. It is well known that as a scaffold protein eIF4G can recruit translation initiation factors to promote translation. However, it can also recruit repression factors to downregulate translation. The latest in this category is a class of RGG-motif repeats containing proteins that bind eIF4G through RGG-motifs to downregulate translation [15]. Since many proteins with RGG-motifs are reported to bind eIF4G (such as Scd6, Sbp1, Ded1, Khd1, Npl3) [15–17], a key question in the field is the mechanism that keeps these repressor proteins under check and allows binding to eIF4G only under specific conditions. It will be imperative to understand these mechanisms since it would provide insights into how eIF4G might transition between different mRNPs leading to translation control. Here we provide the details of one such mechanism using yeast Scd6 (Suppressor of Clathrin Deficiency 6) as a model RGG-motif protein.

**Figure 1.**
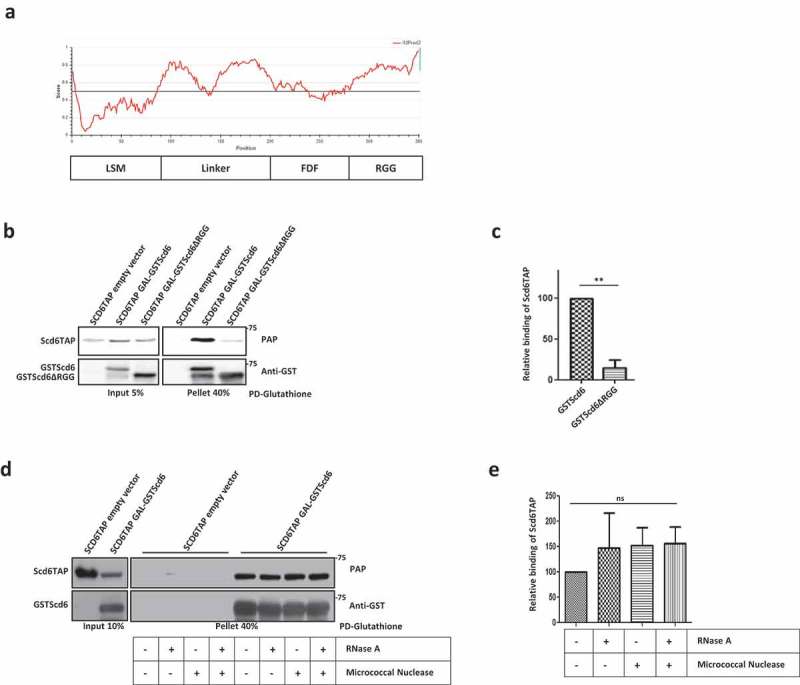
Scd6 binds self *in vivo*. a) Domain organization of Scd6 and analysis of Scd6 sequence to predict intrinsically disordered regions using IUPRED2A. A score above 0.5 indicates disorder in a given region. Entire RGG domain of Scd6 and parts of linker region is predicted to be disordered. b) Galactose inducible GSTScd6 and GSTScd6∆RGG was pulled down from yeast cells expressing Scd6TAP, followed by western blotting to check their interaction. Blot was probed with PAP antibody which recognizes the TAP tag, followed by stripping and anti-GST staining. c) Represents quantitation of three experiments performed as described in b (p=0.009). d) Galactose inducible GSTScd6 was pulled down from cells expressing Scd6TAP in the presence of RNase a, Micrococcal nuclease or both. Samples were analyzed by western blotting and stained as in b. e) Represents quantitation of three experiments performed as described in d.

**Figure 2.**
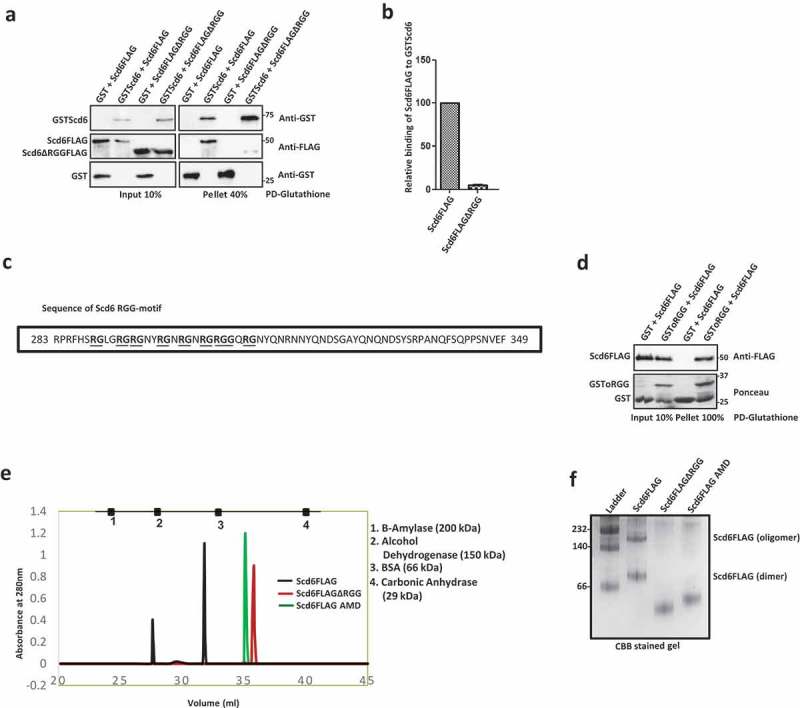
Scd6 directly binds self *in vitro*. a) Recombinant purified Scd6FLAG or Scd6FLAGΔRGG was incubated with purified GSTScd6 followed by Glutathione pull down and western blotting with anti-GST and anti-FLAG antibody. b) Quantitation of two independent experiments performed as in a. c) Sequence of RGG-motif of Scd6 comprising seven ‘-RG’ and one ‘-RGG’ repeats. d) Recombinant purified Scd6FLAG was incubated with purified GSTScd6oRGG or GST alone followed by Glutathione pull down. Scd6FLAG was analysed by western blotting using anti-FLAG antibody and GST/GSToRGG was analysed by Ponceau staining. e) Purified Scd6FLAG, Scd6FLAGΔRGG and Scd6FLAGAMD were run on a gel filtration column (Sephacryl S200 HR) and the figure represents the peak of elution. Gel filtration standard consisted of a mixture of 1. Beta-amylase (200 kDa), 2. Alcohol Dehydrogenase (150 kDa), 3. Bovine Serum Albumin (66 kDa) and 4. Carbonic Anhydrase (29 kDa). f) Purified Scd6FLAG, Scd6FLAGΔRGG and Scd6FLAG AMD were analysed for oligomerization by native PAGE analysis follwed by staining with Coomassie brilliant blue (CBB) staining. GE HMW marker was used as standard. (All recombinant proteins used in this figure are purified from *E.coli*).

Scd6 contains an N-terminal Lsm domain, a central FDF domain and a C-terminal RGG-motif rich domain [18]. RGG-motifs could be defined as repeats of RGG-/RGX-/RG- sequences [19]. The RGG-motif of Scd6 contains 7 RG- and 1 RGG- repeat (Figures 1a & 2c). Scd6 binds eIF4G1 via its RGG-motif, which is augmented by arginine methylation and upon binding eIF4G1, it inhibits the formation of the 48S complex leading to translation repression [15,20]. Scd6 is a conserved translation repressor identified in almost all commonly studied model organisms [21]. Although the mechanism of action has only been elucidated in yeast, evidence from *Arabidopsis, Xenopus* and Humans clearly indicate its role in translation repression [22–24]. The RGG-domain is present in all known Scd6 orthologs except *Plasmodium*. Interestingly orthologs in all higher metazoans such as worms, plants, *Xenopus*, mice and humans have increased number of RGG repeats as compared to yeast Scd6 and these repeats are distributed in two RGG-domains (RGG1 and RGG2) flanking the FDF domain [21]. The functional relevance of overall increased but non-contiguous RGG repeats is unclear, however, it can be hypothesized that such an arrangement highlights the importance of this domain. Consistent with this idea, both RGG-motifs are required for localization of hRAP55/LSM14 (human ortholog of Scd6) to RNA granules [23,25].

Using yeast Scd6 as a model protein, we address how the binding of RGG-domains to eIF4G1 is regulated. We provide compelling evidence suggesting that RGG-domains self-associate, which in turn competes with binding eIF4G1, thus regulating this interaction. We further observe that self-interaction is curtailed upon arginine methylation. Another RGG-motif protein Sbp1 also self-associates through RGG-motif repeats indicating that self-association of RGG-domain repressors could be a new paradigm in translation control.

## Materials and methods

### Yeast strains and plasmids

All strains and plasmids used in this study are listed in the Supplementary Table 1 and 2 respectively. Yeast strains used in this study are BY4741 (wild type) or its derivatives. Strains were grown on either the standard yeast extract/peptone medium (YP) or the synthetic medium (SC) supplemented with appropriate amino acids and 2% glucose or sucrose or galactose (when required). For galactose induction, strains were grown in the presence of sucrose at 30ºC until OD_600_ reached 0.35–0.5. Cells were then pelleted and washed with 2% galactose containing media followed by induction for 12 hours in case of pulldown experiments from yeast cells. Strains were grown at 30ºC until OD_600_ reached 0.35–0.5 for microscopy experiments.

### Protein purification

To purify recombinant HisScd6FLAG and mutants, the protein was expressed in *E.coli* BL21 cells. Cells were induced with 1mM IPTG for 4 hours at 37°C. Cells were lysed in a buffer containing 20mM HEPES-KOH (pH7.6), 150mM KCl, 10% Glycerol, 20mM Imidazole and 1mM β-ME (Buffer C). The lysate was sonicated and clarified in a Beckman JA25.5 rotor at 20,000 rpm for 30 minutes at 4°C. The clarified lysate was passed through a 0.45 µm filter and loaded on an equilibrated HisTrap HP column (GE Healthcare Life Sciences). The protein was eluted with the buffer containing 20mM HEPES-KOH (pH7.6), 300mM KCl, 10% Glycerol, 500mM Imidazole and 1mM β-ME. The protein containing fractions were pooled and carefully diluted with the buffer without KCl. The diluted protein was loaded on an HiTrap Heparin HP column (GE Healthcare Life Sciences) and eluted with a linear gradient from 0 to 100% high salt buffer (0.1 to 1M KCl). The protein fractions were pooled and concentrated in storage buffer containing 20mM HEPES-KOH (pH7.6), 100mM KOAc, 10% Glycerol and 2mM DTT.

GST, GSTScd6, GSToRGG and GSTeIF4G1 were purified from BL21 cells after induction with 1mM IPTG for 4 hours at 37°C. Cells were lysed in a buffer containing 20mM Tris-HCl pH8, 300mM NaCl, 2mM DTT and 1mg/ml Lysozyme. The lysate was sonicated and clarified at 15,000rpm for 15 minutes at 4°C. The clarified lysate was incubated with glutathione sepharose (GE, catalogue no. 17,075,605) for 1 hour. Beads were washed thrice with buffer containing 20mM Tris-HCl pH8, 500mM NaCl and 2mM DTT. Proteins were eluted with buffer containing 20mM Tris-HCl pH8, 150mM NaCl, 2mM DTT and 20mM reduced glutathione.

HisSbp1FLAG and HisSbp1GFP proteins were purified from XL1-Blue cells after induction with 1mM IPTG for 4 hours at 37°C. Cells were lysed in a buffer containing 50mM NaH_2_PO_4_ pH8, 300mM NaCl, 10mM Imidazole, 1mM DTT and 1mg/ml Lysozyme. The lysate was sonicated and clarified at 15,000rpm for 15 minutes at 4°C. The clarified lysate was incubated with Ni-NTA agarose (Thermo Fisher Scientific, catalogue no. 88222) for 1 hour. Beads were washed thrice with buffer containing 50mM NaH_2_PO_4_ pH8, 300mM NaCl, 20 to 50mM Imidazole. Proteins were eluted with buffer containing 50mM NaH_2_PO_4_ pH8, 300mM NaCl, 500mM Imidazole. To remove RNA that might provide bridging interactions, all the extracts were treated with RNase A (10ug/ml). Purified proteins were concentrated and dialyzed into 10mM Tris-Cl pH7, 100mM NaCl, 10% glycerol and 1mM DTT.

TIF4631 (eIF4G1) was sub-cloned in pET28a using NcoI and HindIII sites from pET-Duet eIF4G.eIF4E plasmid [26]. The recombinant His-eIF4G1 protein was expressed in BL21 (DE3) cells by induction with 0.4 mM IPTG at 16°C for 16 hours. The protein was purified using Ni-NTA affinity chromatography followed by Q sepharose ion-exchange chromatography step as described in the above reference [26]. The purified protein was stored in a buffer containing 20mM HEPES-KOH pH7.6, 100mM KOAc pH7.6, 20% glycerol, 2mM DTT at −80°C. Representative CBB stained gel image for all the purified proteins is given in Supplementary Figure 1C.

### Pull down assays

For performing pull-downs from yeast, cells were grown and induced as indicated above. Briefly, cells from a 15ml galactose-induced culture were broken open in 200ul lysis buffer containing 50mM Tris-Cl pH7.5, 50mM NaCl, 2mM MgCl_2_, 0.1% Triton-X100, 1mM β-Mercaptoethanol, RNase A (0.25mg/ml), 1× Complete mini-EDTA-free tablet (Roche, catalogue no. 04693132001) and lysed by vortexing at 4°C in bead-beater with glass beads. Unbroken cells and debris were removed by centrifugation at 5500rpm for 5 min at 4°C, followed by a 2 min spin at 14,000 rpm to remove any protein aggregates. After removing the input sample, the supernatant (200µg protein) was nutated for 2h at 4°C with 30ul of Glutathione Sepharose-4B (GE Healthcare) in 1ml reaction mix with BufferA (containing 50mM HEPES pH7, 100mM NaCl, 1mM DTT, 2mM MnCl_2_, 2mM MgCl_2_, 1% Triton-X100, 10% glycerol, 10mg/ml BSA and RNase A 0.25mg/ml). Beads were washed 3 times (10 min each) with wash BufferA. 10µl of SDS-PAGE loading dye was added to beads and analyzed by SDS-PAGE and western blotting. Micrococcal nuclease was used (2000 units/ml) where indicated.

For recombinant protein pull-downs, 450 nM of purified proteins were incubated with 25 ul of glutathione sepharose beads (GE Healthcare) at 4°C for binding reactions (for 2h) or FLAG-agarose beads (Sigma) in binding buffer. For the competition experiments, up to 1350nM of GSTScd6 was taken. The binding buffer for Glutathione pull downs contained 50mM HEPES pH7, 100mM NaCl, 1mM DTT, 2mM MnCl_2_, 2mM MgCl_2_, 1% Triton-X100, 10% glycerol, 0.25mg/ml RNase A and 10mg/ml BSA. The binding buffer for FLAG pull downs contained 50mM Tris-HCl pH7.4, 150mM NaCl, 1mM EDTA, 1% Triton-X100, 0.25mg/ml RNase A and 10mg/ml BSA. The beads were washed thrice with binding buffer, 10μl of SDS-PAGE loading dye was added to beads and analyzed by SDS-PAGE followed by Western blotting. For pull-downs with *in vitro* methylated proteins, methylation reaction was performed as reported earlier [20].

### Western analysis

Western analysis was performed using standard protocols. The details of antibodies used in this study are as follows: anti-GST (CST, catalogue no. 2624; 1:1000 dilution), anti-FLAG (Sigma, catalogue no. 1:2000 dilution), anti-His (CST, catalogue no. 2366; 1:1000 dilution), Peroxidase anti-peroxidase (Sigma, catalogue no. P1291; 1:1000 dilution), anti-GFP (BioLegend, catalogue no. 338,001; 1:1000 dilution), anti-eIF4G1 (Cocalico Biologicals; 1:1000 dilution), Mono-methyl arginine antibody (CST, catalogue no. 8711; 1:1000).

### Size exclusion chromatography

Size exclusion chromatography was done manually. A size exclusion column was packed using 45 ml of Sephacryl S-200 HR (Sigma-Aldrich) beads. The void volume (24 ml) of this column was determined by running blue dextran. 1mg of protein (in 100 μl volume) was loaded on the column equilibrated with the buffer containing 20mM HEPES-KOH pH7.6, 100mM KCl and 10% Glycerol. Fractions of 50 μl were collected and the absorbance at 280 nM were recorded. Protein fractions were also analyzed on 12% SDS-PAGE. Gel filtration standard consisted of a mixture of Beta-amylase (200 kDa), Alcohol Dehydrogenase (150 kDa), Bovine Serum Albumin (66 kDa) and Carbonic Anhydrase (29 kDa) bought from Sigma.

### Native PAGE analysis

100nM of the purified protein was mixed with 2X native PAGE loading dye (62.5mM Tris-HCl pH6.8, 25% glycerol 1% Bromophenol Blue) and analyzed on a 6% native PAGE (i.e. without βME and SDS) at pH 7.4. The molecular weight of proteins was analyzed on the basis of the HMW Native Marker (GE Healthcare Life Sciences). For analyzing complex formation by native PAGE, increasing concentration of Scd6 (100nM, 200nM and 400nM) was mixed with 100nM of eIF4G and incubated at 4°C. 2X native PAGE loading dye was added to the complex and analyzed on a 6% native PAGE.

### Microscopy

For all experiments, yeast cultures were grown to OD_600_ of 0.35–0.5 in the appropriate synthetic drop-out media at 30°C. Cells were pelleted and spotted on coverslips for immediate microscopic examination at room temperature. All images were acquired using a Deltavision Elite microscope system running softWoRx 3.5.1 software (Applied Precision, LLC), using an Olympus 100x, oil-immersion 1.4 NA objective. Exposure time and transmittance settings for GFP channel were 0.25 sec and 32% respectively and that for mCherry were 0.4 sec and 50%. Images were collected as 512 × 512-pixel files with a CoolSnapHQ camera (Photometrics) using 2 × 2 binning for yeast. All yeast images were deconvolved using standard softWoRx deconvolution algorithms. ImageJ was used to adjust all images to equal contrast ranges according to the experiment conducted or protein examined. For each experiment, 100–150 cells were counted. Data from three independent experiments were used for quantitation and statistical significance was calculated using t-test. For cycloheximide treatment, cells were grown as above and treated with 100ug/ml cycloheximide for 5 minutes followed by live cell imaging.

### RNA isolation

To check the presence of RNA in the pull down experiment, RNA was isolated from untreated lysate and lysates treated with RNase A and/or Micrococcal nuclease by Hot Phenol method. Briefly, 400ul acidic phenol pH4.5 (0.1M citrate buffer saturated) was added to the lysate and incubated at 65^0^C for 15min. Samples were spun at 14,000rpm for 10min at 4^0^C and the top aqueous layer was taken in a fresh tube. 400ul chloroform was added, mixed and spun as above. The aqueous layer was taken and added with 1/10 volume of 3M NaAc pH5.2 and 2.5 volumes of 100% ethanol and incubated at -80^0^C overnight for precipitation. The samples were spun as above and the pellet was washed with 70%ethanol, dried and resuspended in 50ul nuclease free water. RNA was run on 1.2% formamide agarose gel.

## Results

### Scd6 interacts with itself in RGG-motif dependent manner

Scd6 is a modular protein with an N-terminal Lsm domain, a central FDF domain and a C-terminal RGG-motif rich domain (Figures 1a and 2c). RGG-/RGX- repeats present in the C-terminus represent low complexity sequences, which can lead to disordered regions in a protein. IUPRED analysis [27] of Scd6 indeed predicts the presence of disordered region at the C-terminus (Figure 1a). Interestingly, parts of the linker sequence are also predicted to be disordered. Since the RGG-motif rich domain plays an important role in Scd6 translation repression activity, we decided to focus on it. Low complexity QN-rich sequences can self-interact to form higher order structures [28,29].

We tested if Scd6 can interact with itself in yeast. We performed glutathione pull-downs using yeast strains expressing Scd6TAP (chromosomally tagged) along with either GSTScd6 or GSTScd6∆RGG under galactose-inducible promoter. We observed that Scd6TAP can interact with GSTScd6 as Scd6TAP was detected with full-length GSTScd6 as bait in the pull-down assay. Strikingly, GSTScd6∆RGG was defective in binding Scd6TAP (Figure 1b & c). The RGG-deletion mutant of Scd6 binds RNA albeit not as efficiently as the wild type protein [15] indicating that the Scd6 RGG-motif could contribute to RNA binding. RGG-motif self-association could be influenced by RNA binding. However, the self-interaction was independent of RNA as the yeast lysates were treated with RNase A and the same was also added to pulldown reactions. To further rule out if any residual RNA or DNA was mediating the interaction, yeast lysates were treated with RNase A or Micrococcal nuclease or both (Figure 1d & e). The lysates were checked for the presence of RNA and we confirmed that RNase A/micrococcal nuclease treatment was efficient in degrading cellular RNA (Supplementary Figure 1A). We observed that the treatment did not compromise Scd6 self-association activity (Figure 1d), thereby proving that it is independent of mediation by nucleic acids.

### Scd6 self-associates to form a dimer and an oligomer

To test if the binding of Scd6 with itself was due to a direct interaction, we purified recombinant Scd6FLAG and Scd6ΔRGGFLAG and tested their ability to bind recombinant GSTScd6 by performing glutathione pulldowns. Consistent with pull-downs from cells, Scd6FLAG interacted with full-length GSTScd6 but not, Scd6ΔRGGFLAG (Figure 2a & b). Recombinant GST served as a negative control and it did not bind Scd6FLAG or Scd6ΔRGGFLAG, clearly indicating that Scd6 can self-associate directly and the RGG domain is necessary for self-interaction.

To check if the RGG motif is sufficient for self-interaction, binding of purified full-length Scd6FLAG was tested with GST tagged only RGG (oRGG) motif containing peptide. The RGG motif sequence of Scd6 is shown in Figure 2c. The GSToRGG motif was able to pull down Scd6FLAG as seen in the pellet fraction suggesting that the RGG motif of Scd6 is sufficient for self-association (Figure 2d).

Self-association of Scd6 could result in the formation of a dimer, an oligomer or both. We tested the oligomeric status of the protein *in vitro* using two approaches. We performed size exclusion chromatography using purified Scd6 protein and its RGG-motif deletion mutant. The elution profile of gel filtration standards (consisting of a mixture of Beta-amylase-200 kDa, Alcohol Dehydrogenase-150 kDa, Bovine Serum Albumin-66 kDa and Carbonic Anhydrase-29 kDa) is shown in Figure 2e. The predicted molecular mass of purified Scd6 is about 41.5 kDa. We observe that the monomer form of wild type Scd6 (expected to elute between BSA and Carbonic Anhydrase) was undetectable (Figure 2e). Importantly there are at least two higher forms of Scd6, a possible dimer (eluting between BSA and Alcohol dehydrogenase) and a multimer (eluting between Beta-amylase and Alcohol dehydrogenase). The RGG-deletion mutant (predicted molecular mass of about 34 kDa) did not form a dimer or an oligomer and eluted between BSA and Carbonic anhydrase. These results were confirmed by native PAGE analysis where the Scd6 monomer form, which would migrate below the 66 kDa molecular mass standard band, was not detectable. Instead, it migrated as a possible dimer above the 66 kDa band and as a multimeric form that migrated between the 140 kDa and 232 kDa standard bands (Figure 2f). Scd6ΔRGG did not form detectable dimer or higher order structure (Figure 2f) further confirming the pull-down and size exclusion chromatography results. Interestingly, the arginine methylation defective mutant (AMD) [20], in which nine arginines in the RGG motif have been converted to alanine, failed to form a detectable dimer or an oligomer when analyzed using both size exclusion chromatography and native PAGE (Figure 2e & f). These results highlight the importance of the RGG-motif and arginines in self-association.

### Scd6 localizes to foci distinct from repression foci in absence of stress

**Figure 3.**
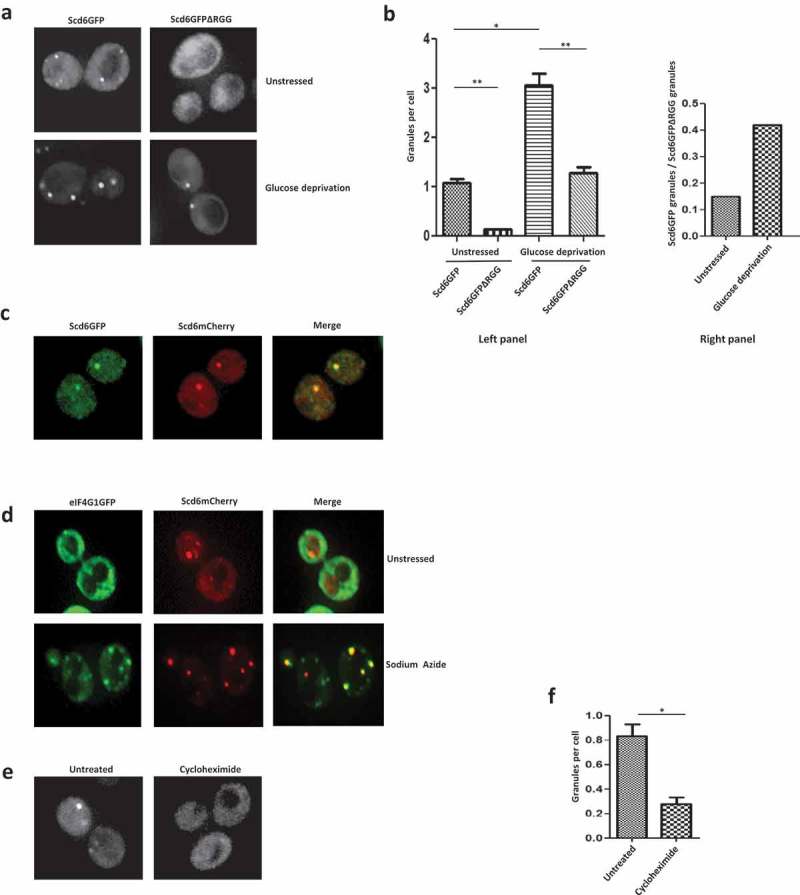
Scd6 localizes to foci in the absence of stress. a) BY4741 cells expressing Scd6GFP and Scd6GFPΔRGG were grown until OD_600_ 0.35–0.5 followed by 10 minute glucose deprivation stress. Live cell imaging was performed for stressed and unstressed cells. b) In the left panel, quantitation of three independent experiments performed as in a has been represented (Scd6GFP Unstressed-Scd6GFPΔRGG Unstressed p=0.0079, Scd6GFP Unstressed-Scd6GFP Stressed p=0.0105, Scd6GFP Stressed-Scd6GFPΔRGG Stressed p=0.0078). In the right panel, the ratio of Scd6GFP granules/cell to Scd6GFPΔRGG/cell in unstressed and glucose deprived condition has been represented. The ratio has been determined based on the quantitation presented in b. c) Scd6mCherry was expressed in SCD6GFP strain, grown until OD_600_ 0.35–0.5 followed by live cell imaging. d) Scd6mCherry was expressed in EIF4G1GFP strain, grown until OD_600_ 0.35–0.5 followed by treatment with 0.5% Sodium Azide for 30 minutes and live cell imaging. e) Scd6mCherry expressing cells were grown until OD_600_ 0.35–0.5 followed by treatment with Cycloheximide(100ug/ml) for 5 minutes and live cell imaging was performed. f) Quantitation of three independent experiments performed as in e (p=0.0191).

A conserved feature of translation repressors is localization to RNA granules such as P-bodies and stress granules in response to stress [30]. In yeast, translation repressor proteins such as Dhh1 and Pat1, are generally present diffused in the cytoplasm under normal growth conditions and relocalize to granules in response to stress. Scd6 localizes to stress granules and P bodies upon stress [15]. Careful analysis using live cell imaging of the plasmid encoded GFP-tagged Scd6 revealed that it localized to foci even in the absence of stress during midlog phase (Figure 3a) and the localization to foci increased in response to glucose deprivation during mid-log phase (Figure 3a & b). Genomically tagged Scd6GFP from a strain expressing Scd6-mCherry also localized to foci in absence of stress (Figure 3c).

To test the role of RGG-motif in Scd6 localization to foci in the absence of stress, an RGG-motif deletion mutant construct was created. We observed that Scd6ΔRGGGFP localized very poorly to foci in the absence of stress (Figure 3a & b). Interestingly this mutant shows some localized signal, the relevance of which remains to be explored. Despite localizing to another organelle-like structure, the overall cytoplasmic signal of this mutant is comparable to that of the wild type which is consistent with the observation that RGG-motif deleted mutant is expressed at higher levels than the wild type protein (Figure 1b, input). Upon stress, localization of wild type Scd6 to foci increased (Figure 3a & b). Although the localization of the RGG-motif deletion mutant in response to stress was defective as compared to the wild type, it still increased (Figure 3a & b). Interestingly, the absence of RGG-motif affects localization to foci more in the absence of stress than in its presence (Figure 3b, right panel).

To test if Scd6 tagged with different fluorophores colocalized, we checked the localization of Scd6mCherry in a strain expressing genomically tagged Scd6GFP and observed that in the absence of stress, 95% of the Scd6GFP foci colocalized with the Scd6mCherry foci (Figure 3c).

Scd6 targets eIF4G1 to inhibit translation initiation [15]. In such a scenario, the Scd6 (repression) foci that arise in response to stress could colocalize with the eIF4G1 foci. The Scd6 foci that arise in the absence of stress could exclude eIF4G1 if these were not Scd6 repression foci. We observed that Scd6mCherry localized to foci as observed earlier in the absence of stress, however, eIF4G1-GFP did not localize to foci under these conditions (Figure 3d). Thus, the foci that arise in the absence of stress contain undetectable amounts of eIF4G1 and do not represent the conventional Scd6 repression foci. To test if these foci contain mRNA, cells were treated with cycloheximide (5 minutes) before observing under the microscope. Significant reduction in the number of foci was visible upon cycloheximide treatment (Figure 3e & f) suggesting that these foci likely contain mRNA. The identity of resident mRNAs and proteins will be a very important future direction. Based on the above result, we propose that the Scd6 foci in absence of stress likely contain self-associated Scd6.

### Self-interaction of Scd6 competes with eIF4G1 binding

**Figure 4.**
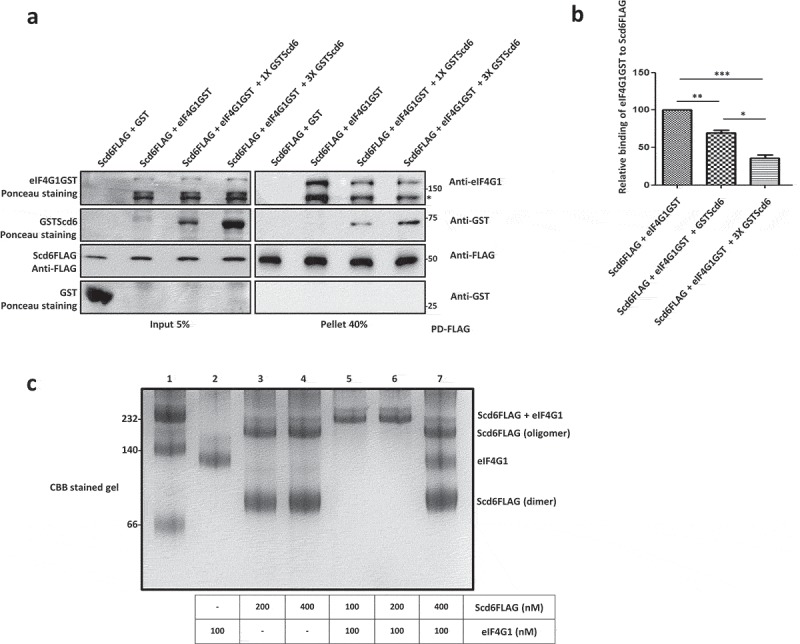
Scd6 self-interaction competes with eIF4G1 binding. a) Purified Scd6FLAG (450nM) was incubated with purified eIF4G1GST (450nM) in the presence of increasing amounts of GSTScd6 (none, 1X-450nM and 3X-1350nM) followed by FLAG pull down. The input blots were stained with Ponceau to visualise eIF4G1GST, GSTScd6 and GST, and anti-FLAG western blotting was performed for Scd6FLAG. Pull down samples were analysed by western blotting with anti-eIF4G1, anti-GST and anti-FLAG (‘*’ represents degradation product of eIF4G1GST). b) Quantitation of three independent experiments performed as in a (none-1X p=0.0032, none-3X p=0.0007, 1X-3X p=0.011). c) 100nM, 200nM and 400nM of Scd6 was mixed with 100nM of eIF4G and incubated overnight at 4°C. 2X native PAGE loading dye was added to the complex and analyzed on native PAGE followed by CBB staining.

We wanted to understand how Scd6 self-association affected its other interactions. For example, Scd6 represses translation by binding to eIF4G1 [15,20] via its RGG motif. Since the RGG motif of Scd6 is also involved in binding itself, it is possible that one interaction could influence another, by either promoting it or competing with it. We hypothesized that self-interaction could be a way of regulating translation repression activity of Scd6 by sequestering it to prevent from binding eIF4G1 when Scd6 mRNA targets need to be translated. Disruption of self-association might aid in the binding of Scd6 to eIF4G1 thereby repressing translation in response to certain physiological cue(s). One of the predictions of this model would be that Scd6 self-interaction could compete with its binding to eIF4G1. To test this, we performed FLAG pull downs and checked the ability of recombinant eIF4G1 to bind purified recombinant Scd6FLAG in the presence of increasing amounts of recombinant GSTScd6 (none, 1X and 3X; Figure 4a). Recombinant GST served as a control. We observed that the ability of Scd6-FLAG to bind eIF4G1 is significantly compromised in the presence of even 1x concentration of GSTScd6 and it further goes down upon increasing the concentration of GSTScd6 (Figure 4a and b). Concomitant with the decrease in eIF4G1 binding to Scd6-FLAG, the binding of GSTScd6 to Scd6-FLAG was clearly visible in the 1X lane which further increased in 3X lane indicating that the self-association of Scd6 was competing with eIF4G1 binding.

In a parallel approach, we incubated increasing amounts of recombinant Scd6FLAG (100 to 400nM) and eIF4G1 (100nM) in Buffer C and allowed them to form a complex. These samples were analyzed by native PAGE to check the effect of increasing concentrations of Scd6 on the Scd6-eIF4G1 complex formation (Figure 4c). Scd6FLAG and eIF4G1 alone were loaded as controls (lane 2 to 4). We observed that at 1:1 and 2:1 ratio of Scd6:eIF4G1, there is a complex formation evident by the appearance of a higher molecular weight band in lane 5 and 6. But in the presence of higher concentrations of Scd6FLAG (4:1 ratio of Scd6:eIF4G1), we observed that the complex formation is inhibited and there is an evident reappearance of Scd6 dimer and oligomer bands, as well as the eIF4G1 band (lane 7). These results clearly indicate that the Scd6 self-interaction competes with the Scd6-eIF4G1 interaction.

### Arginine methylation directly hampers Scd6 self-association

**Figure 5.**
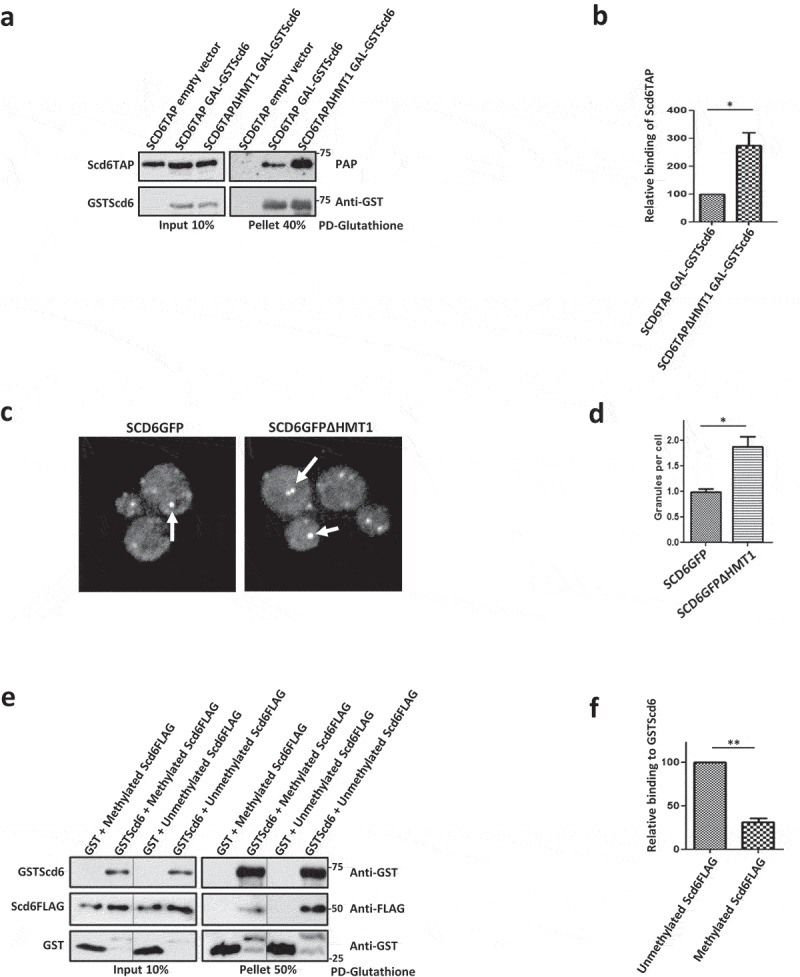
Methylation curtails Scd6 self-interaction a) GSTScd6 was pulled down from Scd6TAP expressing wild type and ∆HMT1 cells. Samples were analysed by western blotting with PAP antibody followed by stripping and anti-GST staining. Empty vector served as control. b) Quantitation of three independent experiments performed as in A (p=0.029). c) Localization of Scd6GFP to foci was checked in SCD6GFP and SCD6GFP∆HMT1 strains expressing Scd6mCherry. Cells were grown until OD_600_ 0.35–0.5 followed by live cell imaging. d) Quantitation of three independent experiments performed as in c (p=0.045). e) Recombinant purified *in vitro* methylated and unmethylated Scd6FLAG was incubated with GST and GSTScd6 followed by Glutathione pull down and western blotting with anti-GST and anti-FLAG antibody to check their interaction (A lane has been omitted in the blots between second and third lane). Cross-reactivity to GST antibody observed in lanes 2 and 4 was due to breakdown of GSTScd6. f) Quantitation of three independent experiments performed as in e (p=0.0017).

RGG-motif of Scd6 is the site of self-association (Figures 1 & 2), eIF4G1 binding [15] and arginine methylation [20]. We wondered if arginine methylation affected the ability of Scd6 to self-associate and tested this possibility both *in vitro* and in yeast cells. We compared the ability of GSTScd6 to associate with Scd6TAP in the wild type and the hmt1Δ background. Hmt1 (hnRNP methyltransferase 1) is the predominant arginine methyltransferase in yeast, which has been shown to methylate Scd6 and promote its repression activity [20]. We observed increased self-association of Scd6 in the absence of Hmt1 (Figure 5a & b) indicating that arginine methylation negatively affects the self-association of Scd6.

Scd6 localizes to foci in the absence of stress that lack detectable eIF4G1 and perhaps contain self-associated Scd6. Since the absence of Hmt1-mediated methylation increased self-association, we tested if the localization of Scd6 to granules in the absence of stress was affected by hmt1Δ background. We looked at the localization of genomically tagged Scd6GFP to foci in the cells expressing plasmid encoded Scd6-mCherry. In the absence of stress, we observed increased localization of Scd6GFP to foci in Δhmt1 background (Figure 5c & d). Above results put together indicate that the self- association of Scd6 is negatively affected by arginine methylation.

The possible mechanism by which methylation reduces Scd6 self-association could be direct or mediated by changes in other cellular interactions in the absence of Hmt1. To differentiate between these possibilities, it was imperative to test the direct effect of methylation on Scd6 self-interaction. We methylated purified recombinant Scd6FLAG *in vitro* by incubating with Hmt1 and SAM. In control reaction, Scd6FLAG protein was incubated with Hmt1 in absence of SAM. Methylation of Scd6 was confirmed based on staining using mono-methylarginine specific antibody (8711, CST). Cross-reactivity with Scd6 was observed only when incubated with Hmt1 in presence of SAM (Supplementary Figure 1B). We observed that the ability of Scd6 to interact with self was reduced upon methylation (Figure 5e & f). This result confirms that Scd6 self-association is directly hampered by its methylation and is consistent with the observations in yeast cells (Figure 5a–d). Since methylation decreases Scd6 self-interaction and increases Scd6 interaction with eIF4G1, we propose that it acts as a kind of switch to regulate Scd6 role in repression.

### Sbp1 self-associates in RGG-motif dependent manner

**Figure 6.**
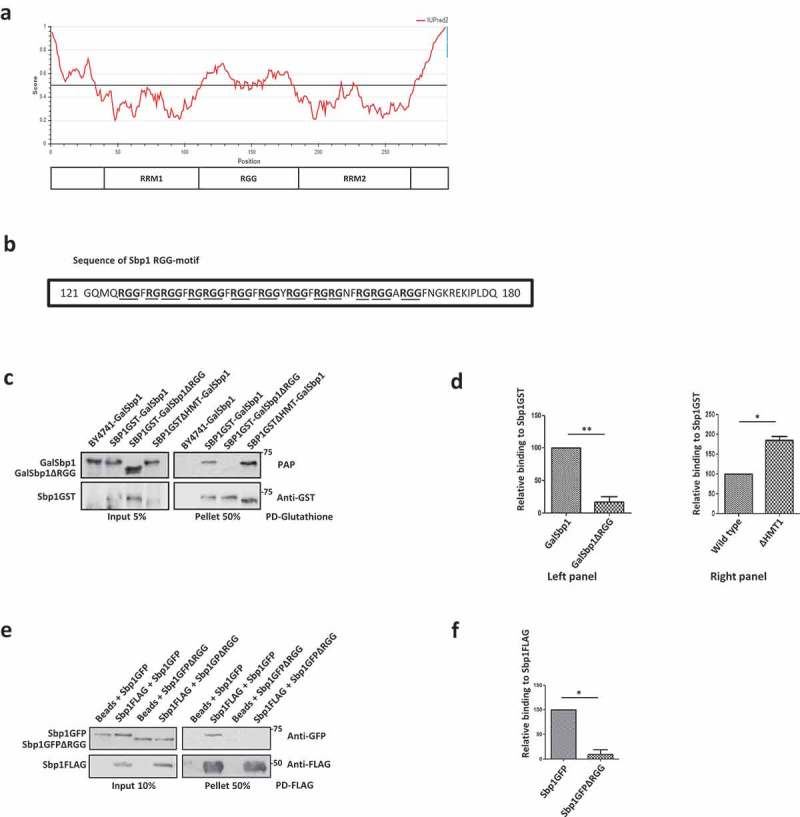
Sbp1 binds self in RGG-motif dependent manner. a) Domain organization of Sbp1 and analysis of Sbp1 sequence to predict intrinsically disordered regions using IUPRED2A. A score above 0.5 indicates disorder in the region. Entire RGG domain of Sbp1 and the termini adjacent to RRM domains are predicted to be disordered. b) Sequence of RGG-motif of Sbp1 comprising five ‘-RG’ and eight ‘-RGG’ repeats. c) Sbp1GST was pulled down from wild type yeast cells expressing galactose-inducible Sbp1/Sbp1∆RGG and galactose-inducible Sbp1 from ∆HMT1 cells. BY4741 (without Sbp1GST) expressing Gal-Sbp1 acted as a control. Samples were analysed by western blotting with anti-GST followed by stripping and PAP antibody staining. d) Represents quantitation of three experiments performed as described in C. Quantitation was done separately for comparing wild type to Sbp1∆RGG (p=0.009, left panel) and wild type to ∆HMT1 (p=0.015, right panel). e) Recombinant purified Sbp1FLAG was incubated with purified Sbp1GFP and Sbp1GFPΔRGG followed by FLAG pull down and western blotting with anti-GFP and anti-FLAG antibody. f) Represents quantitation of three experiments performed as described in E (p=0.0111).

We next addressed if self-association mediated by the RGG-motif could be a general property of RGG-motif containing repressor proteins. We decided to focus on Sbp1, which is another translation repressor that binds eIF4G1 [15,31,32]. IUPRED analysis of Sbp1 sequence predicted the RGG-motif region to be disordered (Figure 6a) whereas the RRM domains were expectedly predicted to be folded. We first looked at the self-association of Sbp1 in cells. Glutathione pull-downs were performed from chromosomally-encoded SBP1GST strain expressing Sbp1 or Sbp1ΔRGG on plasmids. We observed that Sbp1 associated with itself and this association was dependent on the RGG-motif since the Sbp1ΔRGG mutant failed to interact (Figure 6c & d).

Sbp1 is also reported to be arginine methylated in its RGG motif [33]. Hence, we tested if arginine methylation could affect Sbp1 self-interaction as in the case of Scd6. We compared the self-binding of Sbp1 in wild type and Δhmt1 background. Like Scd6, Sbp1 self-association was elevated in the absence of arginine methylation suggesting that arginine methylation curtails self-interaction of the RGG-motif protein (Figure 6c & d).

To test if the self-association was direct, we purified wild type and RGG-deletion mutant in recombinant purified form. Upon performing pull-downs using recombinant purified proteins in the presence of RNase A, we observed that full-length Sbp1FLAG binds itself. It interacts with Sbp1GFP, but the mutant lacking RGG-motif did not, suggesting that the direct interaction of Sbp1 with itself is dependent on its RGG-motif (Figure 6e & f). Like Scd6, Sbp1 localizes to P bodies and stress granules when cells are subjected to stress [34]. We wanted to test if Sbp1 is also capable of forming foci under unstressed condition which may harbour self-associating form of the protein. We performed live cell imaging with cells expressing genomically tagged Sbp1GFP expressed under its own promoter in log phase (OD_600_ 0.35–0.5). We observed that Sbp1 localizes to foci in the absence of stress (Supplementary Figure 1D). Our results with Sbp1 clearly indicate that it self-associates in RGG-motif dependent manner in cells and *in vitro*. Further like Scd6, self-association is modulated by methylation.

## Discussion

The experimental results presented here establish the role of RGG-motif rich domains in self-association and identify a crucial role of self-association in regulating the eIF4G-Scd6 interaction. Our results also provide evidence for the role of arginine methylation in regulating self-association of Scd6.

Several results point towards the contribution of RGG-motif to self-association: a) Pull-downs from yeast cells and using purified proteins, establish that Scd6 binds itself in RGG-motif dependent manner (Figure 1b,c, 2a & 2b), b) Self-association is independent of RNA/DNA since the treatment with RNase A/nuclease does not affect this interaction (Figure 1d & e), c) Scd6 RGG-motif is sufficient for binding itself, highlighting the direct role of RGG motif in binding itself (Figure 2d), d) Size exclusion chromatography and native PAGE analysis indicate that Scd6 can form a dimer and an oligomer (Figure 2e & 2f) e) Arginines play an important role in self-association as substitution of arginines with alanine in the RGG-motif of Scd6 rendered the protein defective in forming dimer and oligomer (Figure 2e & f), f) Sbp1, another eIF4G1 targeting RGG-motif protein, self-associates both in cells and *in vitro* in RGG-motif dependent manner (Figure 6a–e).

Competition between Scd6 self-association and eIF4G binding is indicated through the result where an increased concentration of Scd6 disrupts the Scd6-eIF4G interaction as observed by pull-downs and native PAGE experiments (Figure 4a–c). Finally, we uncover the role of arginine methylation in negatively regulating self-association. We observed that through following results a) recombinant purified methylated Scd6 interacts with itself weaker than the unmethylated Scd6 (Figure 5e & f), b) self-association of Scd6 increases in the absence of Hmt1 in yeast (Figure 5a & b). These results indicate that methylation switches interactions of Scd6 allowing it to transition from a self-associated state to a eIF4G1-bound state that represses translation.

Our results with Scd6 point towards the possibility that RGG domains might function as self-association domains in a number of proteins. Direct self-association of Sbp1 through its RGG-motif (Figure 6c–f) corroborate this idea. RGG-domains contain repeats of RGG-/RGX-/RG- which makes them low complexity sequences [35]. Other low complexity sequences like QN-rich sequences are often found in prions and can bind itself [36]. Although self-association of purified EWS has been reported to be mediated by RGG-motif [37], evidence for its self-association *in vivo* and a possible functional role of EWS self-association remain unclear. In the EWS report since the effect of RNase/nuclease on the self-association of purified EWS proteins has not been tested, it is not clear if the observed self-association is due to two EWS protein molecules binding the same RNA/DNA molecule since the EWS protein is a known RNA-binding protein [38]. The sufficiency of EWS RGG-motif for self-association and the impact of arginine methylation are also unexplored.

Studies done with Scd6 orthologs provide indications both for and against self-association. Purified full length Dcp5, (*Arabipopsis* ortholog of Scd6) interacts with self through RGG-FDF-RGG domain [22]. However, the requirement of RGG for self-association *in vivo* and the significance of this interaction have not been explored. Contrary to *Arabidopsis*, Scd6 orthologs in *Drosophila* (Trailer hitch) and Trypanosome (SCD6) do not associate with self [39,40] (data not shown in both reports). We believe this could be due to differences in the RGG-motif sequence in these orthologs. Interestingly in Tral and SCD6 (as well as in other known higher eukaryotes), the RGG-motif is split due to the presence of the FDF motif leading to two RGG-motif rich sequences RGG1 and RGG2 [21]. Perhaps a critical length of contiguous RGG-/RGX- sequence is required for self-association. Both Tral (6 repeats in RGG1) and SCD6 (4 repeats in RGG2) have fewer RGG-/RGX- motifs in a given contiguous stretch as compared to yeast ortholog (8 repeats). Strikingly, DCP5 reported to interact with itself contains 8 repeats in RGG2. Overall the current report to our knowledge is the first comprehensive demonstration of RGG-motif self-association both *in vivo* and *in vitro*. Further, in this work, the relevance of RGG self-association has been highlighted by its competition with eIF4G. For other RGG-proteins it is possible that self-association might modulate the interaction of RGG with bonafide protein/RNA partners.

An interesting aspect raised by this work that needs further investigation is the nature of interaction(s) that might contribute to the RGG-motif self-association. Arginines from adjacent RGG-boxes are likely to repel each other. However, the property of Arginine and Glycine residues allow them to participate in long range pi-pi interactions [35]. The arginine side chain can also participate in hydrophobic and cation-pi interactions. PDB analysis reveals that the Arginine and Glycine residues make more contacts through the pi-pi interactions as compared to other amino acids [35]. Specifically, such pi-pi interactions can contribute to phase separation of low complexity protein sequences [41] which could involve self-association. Consistent with the role of arginines in self-association, the arginine to alanine (AMD) mutant of Scd6 fails to form a dimer or an oligomer (Figure 2e & f). Whether the RGG-motif mediated self-association could lead to phase separation of Scd6 remains to be addressed. It is unclear at this point if the RGG-motif association would limit to only specific homotypic interactions. Self-association assisted by pi-pi like interactions could likely occur between RGG-motifs from different proteins leading to heterotypic interactions. Whether RGG-motifs from different proteins involved in similar cellular processes could bind each other to regulate function, is an exciting possibility raised by our results.

Results presented here provide evidence for arginine methylation directly affecting Scd6 self-association in a switch-like manner which has not been reported earlier. We propose that under conditions when Scd6 repression activity is not required, it is sequestered in the self-associated form that prevents it from binding eIF4G. Arginine methylation inhibits self-association promoting transition to a form competent to interact with eIF4G, which could lead to repression. Our report adds to the increasing repertoire of cellular processes affected by Arginine methylation. RNA-binding proteins are the largest group of arginine methylated proteins hence the mechanism presented here could be true for other RNA binding proteins. Translation repressor and mRNA export factor Npl3 is shown to self-associate better in the absence of methylation, but there is no evidence for a direct effect of arginine methylation on its self-interaction [42]. The role of Arginine methylation in inhibiting self-interaction is indirectly corroborated by the observations that for proteins such as hnRNPA2 [43] and FUS [44] arginine methylation inhibits phase separation behaviour *in vitro*. Inhibition of phase separation by arginine methylation would predict that it would negatively affect RNA granule assembly. The observation that arginine demethylation promotes the assembly of stress granules [45] is consistent with this idea. Contrary to this is the observation that arginine dimethylation promotes P-body assembly in human cells [46] suggesting that methylation affects RNA granule in distinct ways. It is likely that the impact of arginine methylation on LLPS and RNA granule could be mRNP-specific, determined by constituent RNAs and other post-translational modifications. Identifying the mechanistic basis of differential effects of arginine methylation on LLPS *in vitro* and RNA granule *in vivo* will be an important future direction.

Overall this report brings to fore an exciting and previously unreported mechanism underlying the regulation of eIF4G1-binding and RGG-motif containing translation repressors, Scd6 and Sbp1 through RGG self-association. We believe that such a mechanism could be evolutionarily conserved and our work paves way for understanding similar regulatory mechanisms for RGG-motif containing repressors in higher organisms in diverse cellular processes.
