# Supplementary material for: RGG-motif self-association regulates eIF4G-binding translation repressor protein Scd6
Source: RNA Biol. 2019 Jun 12;16(9):1215–27. doi: 10.1080/15476286.2019.1621623 (PMC6693564; doi:10.1080/15476286.2019.1621623)
Supplement: Supplemental Material [file krnb-16-09-1621623-s001.pdf]

a

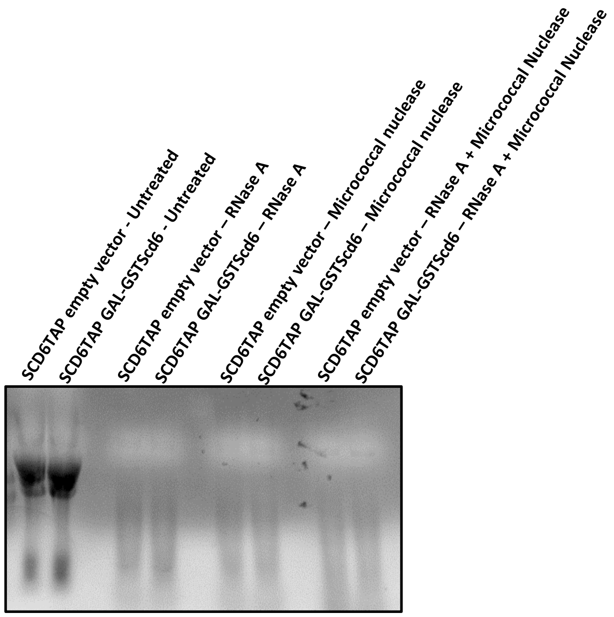

b

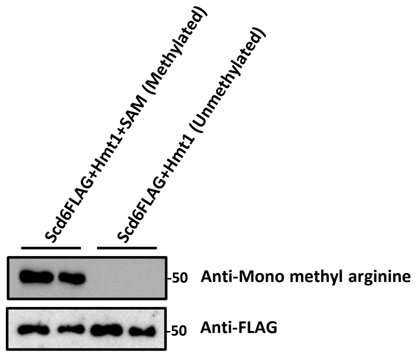

c

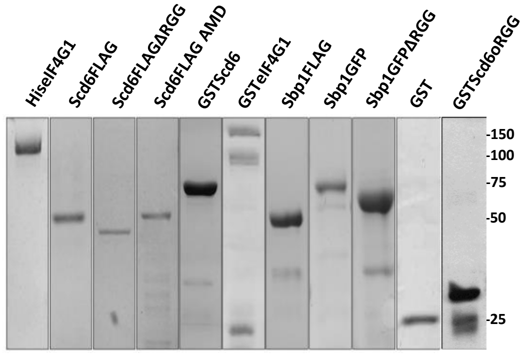

d

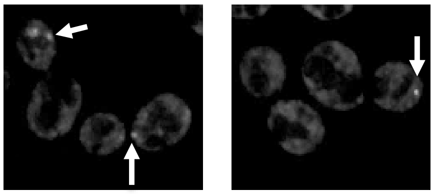

Supplementary Figure 1: a) RNA was isolated from the lysates used for the experiment shown in Fig.1d and ran on 1.2% Formamide-agarose gel to check if the nuclease treatment degraded cellular RNA. b) Recombinant purified Scd6 was *in vitro* methylated using purified Hmt1 and SAM. A part of the reaction was loaded on SDS-PAGE in duplicates followed by western blotting with anti-Mono methyl arginine antibody and anti-FLAG antibody. c) Representative CBB stained gels of purified proteins. d) SBP1GFP expressing cells were grown until OD_{600} 0.35-0.5 followed by live cell imaging. Two representative images have been shown.
