# Supplementary material for: RGG-motif self-association regulates eIF4G-binding translation repressor protein Scd6
Source: RNA Biol. 2019 Jun 12;16(9):1215–27. doi: 10.1080/15476286.2019.1621623 (PMC6693564; doi:10.1080/15476286.2019.1621623)
Supplement: Supplemental Material [file krnb-16-09-1621623-s002.pdf]

Supplementary Table 1: Strains used in this study

Strain Name	Genotype	Source
yPIR1	MATa his3D1 leu2D0 met15D0 ura3D0 ('BY4741')	20
yPIR4	MATa leu2 ura3 his3 met15 eIF4G1-GFP (HIS)	15
yPIR7	MATa his3D1 leu2 ura3 his3 met15 SBP1-GST::KanMX	Bhatter *et al.,* (unpublished)
yPIR8	MATa his3D1 leu2 ura3 his3 met15 SBP1-GST::KanMX hmt1Δ::Hyg	Bhatter *et al.,* (unpublished)
yPIR9	MATa leu2 ura3 his3 met15 Sbp1-GFP (HIS)	Bhatter *et al.,* (unpublished)
yPIR13	MATa leu2 ura3 his3 met15 Scd6-GFP (HIS)	15
yPIR54	MATa leu2 ura3 his3 met15 Scd6-GFP (HIS) hmt1Δ::KanMX	This study
yPIR14	MATa leu2 ura3 his3 met15 Scd6-TAP (HIS)	A kind gift from Roy Parker
yPIR15	MATa leu2 ura3 his3 met15 Scd6-TAP (HIS) hmt1Δ::Hyg	This Study

Supplementary Table 2: Plasmids used in this study

Plasmid Name/number	Description	Vector	Source
pPIR1	Used to express full length Scd6 with His- and FLAG tag at N- and C-terminus respectively	pPROEX1	15
pPIR2	Used to express Scd6ΔRGG with His- and FLAG tag at N- and C- terminus respectively	pPROEX1	15
pPIR3	Used to express full length Hmt1 with His-tag at N-terminus	pET28b	A kind gift from Anita Corbett
pPIR4	Used to express full length GST	pGEX6P3	20
pPIR6	2μ plasmid with gal-inducible promoter used as empty vector control	BG1805	20
pPIR13	2μ plasmid expressing GST-Scd6 under gal-inducible promoter	pEGH	20
pPIR14	2μ plasmid used for expressing Sbp1 under galactose promoter	BG1805	Bhatter *et al.,* (unpublished)
pPIR15	2μ plasmid expressing Sbp1ΔRGG (Δ 125 to 167 amino acid) under gal-inducible promoter	BG1805	Bhatter *et al.,* (unpublished)
pPIR29	Used for expressing Sbp1 with N-terminal 6X His tag and C-terminal FLAG tag cloned using BamH1 and Xho1 restriction site	pPROEX1	Bhatter *et al.,* (unpublished)
pPIR47	Used for expressing full length eIF4G1 tagged N-terminus to GST and C-terminus to 6X-HIS	pGEX6P3	15
pPIR83	Used for expressing full length Scd6 tagged N-terminus to GST and C-terminus to 6X-HIS	pGEX6P3	15
pPIR84	Used for expressing only RGG domain of Scd6 tagged N-terminus to GST and C-terminus to 6X-HIS	pGEX6P3	This Study
pPIR96	2μ plasmid to express Scd6mCherry under its own promoter	pYES	This Study
pPIR93	Cen plasmid to express Scd6GFP under its own promoter	pRS316	This Study
pPIR94	Cen plasmid to express Scd6GFPΔRGG under its own promoter	pRS316	This Study
pPIR57	Used for expressing Sbp1 with N-terminal 6X His tag and C-terminal GFP tag	pPROEX1	This Study
pPIR58	Used for expressing Sbp1ΔRGG with N-terminal 6X His tag and C-terminal GFP tag	pPROEX1	This Study
pPIR85	Used to express Scd6R288A, R292A, R294A, R298A, R301A, R304A, R306A, R310A, R316A with His- and FLAG tag at N- and C- terminus respectively	pPROEX1	This Study
pPIR77	2μ plasmid expressing GST-Scd6ΔRGG under gal-inducible promoter	pEGH	This Study
pPIR103	Used for expressing eIF4G1 with His tag at N and C terminus	pET28a	This Study
